# The risk of viral transmission in feed: What do we know, what do we do?

**DOI:** 10.1111/tbed.13606

**Published:** 2020-07-10

**Authors:** Scott A. Dee, Megan C. Niederwerder, Gil Patterson, Roger Cochrane, Cassie Jones, Diego Diel, Egan Brockhoff, Eric Nelson, Gordon Spronk, Paul Sundberg

**Affiliations:** ^1^ Pipestone Applied Research Pipestone Veterinary Services Pipestone MN USA; ^2^ Department of Diagnostic Medicine/Pathobiology College of Veterinary Medicine Kansas State University Manhattan KS USA; ^3^ Center for Animal Health in Appalachia Lincoln Memorial University Harrogate TN USA; ^4^ Department of Animal Science and Industry Kansas State University Manhattan KS USA; ^5^ Department of Population Medicine and Diagnostic Sciences College of Veterinary Medicine Cornell University Ithaca NY USA; ^6^ Prairie Swine Health Services Red Deer AB Canada; ^7^ Department of Veterinary Science South Dakota State University Brookings SD USA; ^8^ Swine Health Information Center Ames IA USA

**Keywords:** African swine fever, animal feed, China, feed mitigation, swine viral diseases, transboundary

## Abstract

The role of animal feed as a vehicle for the transport and transmission of viral diseases was first identified in 2014 during the porcine epidemic diarrhoea virus epidemic in North America. Since the identification of this novel risk factor, scientists have conducted numerous studies to understand its relevance. Over the past few years, the body of scientific evidence supporting the reality of this risk has grown substantially. In addition, numerous papers describing actions and interventions designed to mitigate this risk have been published. Therefore, the purpose of this paper is to review the literature on the risk of feed (*what do we know)* and the protocols developed to reduce this risk (*what do we do*) in an effort to develop a comprehensive document to raise awareness, facilitate learning, improve the accuracy of risk assessments and to identify knowledge gaps for future studies.

## 1 PART 1: WHAT DO WE KNOW?

### 1.1 Introduction

Effective biosecurity protocols are essential towards protecting the health status of swine farms. In the United States, tremendous resources have been invested to reduce the risk of viral pathogens, such as the entry of porcine reproductive and respiratory syndrome virus into susceptible populations. Protocols including shower in‐shower out, transport sanitation, quarantine and testing of incoming genetics and the filtration of incoming air are commonplace throughout the US swine industry, particularly at the level of the sow farm (Silva, Corbellini, Linhares, Baker, & Holtkamp, 2018). In contrast, prior to the May 2013 entry of porcine epidemic diarrhoea virus (PEDV) into the US swine population (Chen et al., 2014) the role of swine feed as a vehicle for pathogen transport and transmission had not been considered, despite the fact that feed is delivered to swine farms on a daily basis in the absence of any biosecurity protocols. Since the identification of this novel risk factor, scientists across North America have conducted numerous studies to understand its relevance. Therefore, the purpose of this paper is to review the literature on the risk of feed (*what do we know?)* and the protocols that have been developed to reduce this risk (*what do we do?),* in an effort to develop a comprehensive document to raise awareness, facilitate learning and identify knowledge gaps for future studies.

### 1.2 PEDV changes the paradigm

Upon its entry to the United States, PEDV spread rapidly throughout the country at an unprecedented rate (Niederwerder & Hesse, 2018). Following phylogenetic analysis, it was determined that the virus most likely originated from China (Huang et al., 2013). During the initial outbreak, the American Association of Swine Veterinarians, the National Pork Producers Council and the USDA Center for Epidemiology and Animal Health conducted an epidemiological investigation involving porcine epidemic diarrhoea (PED) affected case and control herds. Of the more than 100 variables surveyed during the investigation, seven were significantly associated with acquiring PEDV during the process of feeding animals, including using sow feed that was custom mixed off‐farm in the last 90 days prior to the questionnaire, how many meal/mash rations were fed to nursery or finishers in the last 90 days, the total number of different rations fed to finisher pigs in the last 90 days, the contents in terms of supplementation that was in the premix for the most recent finisher diet and what type of grain mix was used for sow or finisher feed in the past 90 days (AASV, NPPC & USDA CEAH, 2015). In 2014, the risk was confirmed when ingestion of contaminated complete feed was proven to be a vehicle for PEDV transmission to naïve pigs (Dee, Clement, et al., 2014). This study involved the detection of viral nucleic acid in feed dust samples from the interior walls of feed bins that had provided feed for the index cases of PED in sows across a subset of farms, followed by a demonstration of virus viability in a pig bioassay model through natural feeding behaviour (Dee, Clement, et al., 2014). Within 3–4 days after consumption, evidence of PEDV infection was noted, including clinical signs of PED (vomiting and diarrhoea), detection of PEDV RNA in rectal swabs and PED‐consistent lesions in the gastrointestinal tract. This publication resulted in a series of laboratory experiments to validate the results and expand upon the concept of feed and feed ingredients as risk factors for viral transport and transmission. It must be noted that while the original study (Dee, Clement, et al., 2014) used naturally contaminated feed, the majority of these follow up experiments involved purposeful inoculation (spiking) of ingredients.

Following the proof of concept study, the minimum infectious dose of PEDV in feed was determined to be 5.6 × 10^1^ TCID_50_/g using the 10‐day‐old piglet bioassay (Schumacher et al., 2016). In addition, the potential for widespread PEDV contamination of surfaces in an animal food manufacturing facility was evaluated (Schumacher et al., 2017). In this study, a U.S. virulent PEDV isolate was used to inoculate 50 kg of swine feed, which was then mixed, conveyed and discharged into bags using pilot‐scale feed manufacturing equipment. Subsequent collection of environmental swabs demonstrated widespread distribution of virus via feed dust, with the presence of PEDV ribonucleic acid (RNA) in 100% of dust samples collected from animal food‐contact surfaces and in 89% of dust samples from non‐animal food‐contact surfaces. Once contamination of the feed mill environment was demonstrated, the question of whether viral survival would differ across the various feed ingredients found in a milling environment was investigated (Dee et al., 2015). A subset of feed ingredients used in swine rations were inoculated with PEDV and stored outdoors during the month of January in Minnesota. Interestingly, viable PEDV was detected by virus isolation or swine bioassay out to 180 days post‐inoculation (DPI) in conventional (high protein/low fat) soybean meal, as well as out to 30 DPI in DDGS, meat and bone meal, RBCs, lysine HCL, D/L methionine, choice white grease, choline chloride, and out to 7 DPI in limestone and 14 DPI in threonine. In contrast, viable PEDV was not present in several other ingredients, including corn, various animal protein sources and vitamin/trace mineral mixes (Dee et al., 2015).

These data, along with observations from the field, posed the question of whether certain ingredients could serve as vehicles for the movement of PEDV between countries. This issue was raised in January 2014, when PEDV was detected for the first time in Ontario, Canada (Pasma, Furness, Alves, & Aubry, 2016). Following extensive epidemiologic investigation, the source of the virus appeared to be contaminated samples of spray‐dried plasma protein originating from the United States (Perri, Poljak, Dewey, Harding, & O'Sullivan, 2018, 2019). However, while infectious PEDV was demonstrated in samples of case‐specific plasma by swine bioassay, transmission of the virus to pigs following consumption of feed containing PEDV‐positive plasma was not successful (Pascik et al., 2014).

Building on the potential for transboundary movement of PEDV, a Trans‐Pacific model, simulating the movement of cargo from Beijing, China, to the Anquing terminal in Shanghai, China to the port of San Francisco, CA, US, and then to Des Moines, IA, US, was developed (Dee et al., 2016). The model utilized transport times, environmental conditions and feed ingredients representative of cargo transport from China to the United States that were purposefully inoculated with virus. Under the conditions of this study, PEDV survived the trans‐oceanic simulation in soy‐based products, lysine, choline and vitamin D (Scott et al., 2016). Surprisingly, the virus did not survive in the absence of a feed matrix, suggesting that survival is dependent upon the presence of the ingredient, not the container (tote) per se.

### 1.3 Expanding the viral portfolio

Based on these collective data involving PEDV, the transboundary experiment was repeated across 11 other viruses, including Senecavirus A (SVA), a surrogate for foot‐and‐mouth disease virus (FMDV), bovine viral diarrhoea Virus (BVDV), a surrogate for classical swine fever virus (CSFV), African swine fever virus (ASFV), influenza A virus of swine (IAV‐S), bovine herpesvirus type 1 (BHV‐1), a surrogate for pseudorabies virus (PRV), canine distemper virus (CDV), a surrogate for Nipah virus (NiV), porcine reproductive and respiratory syndrome virus (PRRSV), porcine sapelovirus, (PSV) a surrogate for swine vesicular disease virus (SVDV), vesicular stomatitis virus (VSV), porcine circovirus type 2 (PCV2), and feline calicivirus (FCV), a surrogate for vesicular exanthema of swine virus (VESV) (Dee et al., 2018).

Under the conditions of this study, SVA (representing FMDV), FCV (representing VESV), BHV‐1 (representing PRV), PRRSV, PSV (representing SVDV), ASFV and PCV2 maintained infectivity as determined by virus isolation or swine bioassay, while BVDV (representing CSFV), VSV, CDV (representing Nipah Virus) and IAV‐S did not. Since ASFV had not been reported in China at the time of the study and was actively circulating in Eastern Europe, a Trans‐Atlantic model was developed involving representative feed ingredients, transport times and environmental conditions simulating shipment from Warsaw, Poland, to the port of Le Havre, France, to the port of New York City, NY, US, and then to Des Moines, IA, US (Dee et al., 2018). As before, following purposeful inoculation of ingredients, the majority of viruses survived in conventional soybean meal, lysine hydrochloride, choline chloride, vitamin D and pork sausage casings. These collective results led to the development of the concept of ‘high‐risk combination’ (i.e. specific ingredients promote the stability of certain viruses) and contributed to the growing body of evidence that contaminated feed ingredients may represent a risk for the transport of pathogens at domestic and global levels.

These findings became significant following the announcement of the initial cases of ASFV in China's pig population during August 2018 for several reasons:

1. Fifty per cent of the world's pigs were in China at the time.
2. The Chinese national herd was naïve to ASFV.
3. Purposeful inoculation of ASFV in 12 feed ingredients resulted in the survival of the virus in nine of the ingredients, including three varieties of soy‐based products, choline chloride, three types of pet food, pork sausage casings and complete feed (Dee et al., 2018).
4. The United States imported approximately 2 M metric tons of agricultural products, including 55,000 metric tons of soy‐based products from China, along with 45,000 metric tons and 3,000 metric tons of soy‐based products from the Ukraine and Russia, respectively, in 2018.

The ASFV outbreak in China accelerated the research efforts to better understand the risk of feed, specifically as it pertained to ASFV. This resulted in the work of Niederwerder and others who documented transmission of ASFV to naïve pigs following natural consumption of purposefully contaminated feed and liquid (Niederwerder et al., 2019). This study determined the minimum infectious dose of ASFV in liquid (10^0^ TCID_50_) and in feed (10^4^ TCID_50_) following a one‐time exposure. However, further analysis indicated that the more frequent the exposure (3×, 10×, 30×) to ASFV in small volumes of feed or liquid, the higher the probability of infection, even in the presence of lower doses such as 10^2^ TCID_50_. Another significant finding was the calculation of ASFV half‐life in feed ingredients. Original estimates based on limited (*n* = 2) data points derived from the trans‐Atlantic model indicated that half‐life ranged from 4.1 to 5.1 days across all nine of the virus‐positive ingredients (Dee et al., 2019). In subsequent work, Niederwerder and others again used the Trans‐Atlantic model to conduct a more comprehensive half‐life evaluation, incorporating data from all four sampling points in the model and increasing the number of replicates. This work resulted in half‐life values that ranged from 9.6 to 14.2 days across all nine supportive ingredients following purposeful inoculation (Stoian et al., 2019), suggesting that ASFV survival could occur far beyond the 30‐day transport period used in the model.

In conclusion, there appears to be a growing body of experimental evidence that specific viruses in combination with the proper ingredient can survive long‐distance transport under simulated transboundary conditions. This evidence corroborates field observations, suggesting that the spread of PEDV had occurred in novel ways, despite the fact that multiple interventions were already in place. This may also be the case for ASFV, although at this time we have no evidence of this is North America. It is now clear that under experimental conditions, pathogens such as PEDV and ASFV can be transmitted through feed, and the minimum oral infectious doses have been calculated. Finally, in further support of feed as a risk factor, a recent publication by Stoian and others documented that following purposeful inoculation of the actual viruses, PRV and CSFV survived throughout the Trans‐Pacific model in feed ingredients (Stoian et al., 2020). Surprisingly, viable PRV was recovered from 9 ingredients (conventional soybean meal, organic soybean meal, lysine, choline, vitamin D, moist and dry pet food, and pork sausage casings) while in contrast, viable CSFV was only recovered from conventional soybean meal and pork sausage casings.

## 2 PART 2: WHAT DO WE DO?

Since the discovery of PEDV in the United States and the role that feed may have played in the epidemiology of the disease, there has been extensive effort put forth to evaluate the efficacy of multiple protocols and products to reduce risk. Reviewing the literature, current publications have centred on one of four approaches: mechanical reduction (flushing and sequencing of feed batches), heat treatment, chemical mitigation and/or storage time of feed. As a complement to this work, a validated sampling method has been developed (Jones, Stewart, Woodworth, Dritz, & Paulk, 2019). This publication indicated that the sampling of bulk ingredients for PEDV should include compositing of least 10 individual samples and that the ability to detect is dependent upon dose and loss of viral load (~10 Ct) during the extraction methods involving feed.

### 2.1 Strategy 1: Mechanical reduction (flushing and sequencing)

Several experiments have been conducted to assess the efficacy of decontaminating feed and feed manufacturing facilities through the physical process of mixing, using repeated sequencing of clean feed following known contaminated batches or through the use of chemically treated rice hulls (Gebhardt et al., 2018; Schumacher et al., 2018) In regards to sequencing, results demonstrated that sequenced batches of feed had reduced quantities of PEDV RNA, although sequenced feed without detectible PEDV RNA was still infectious (Schumacher et al., 2018). Therefore, this protocol can reduce but not eliminate the risk of producing infectious PEDV carryover from the first batch of feed. In regards to the use of chemically treated rice hulls, flushes treated with formaldehyde or medium‐chain fatty acid (MCFA) blends reduced the quantity of detectible RNA present after mixing a batch of PEDV‐positive feed (Gebhardt et al., 2018).

### 2.2 Strategy 2: Heat treatment

Several studies have demonstrated a positive effect of temperature on the inactivation of PEDV in feed (Cochrane et al., 2017; Gerber et al., 2014; Trudeau et al., 2016; Trudeau, Verma, Sampedro, et al., 2017; Trudeau, Verma, Urriola, et al., 2017). Early work on the effect of heat treatment by Trudeau et al indicated that heating swine feed at temperatures over 130°C effectively reduced PEDV survival (Trudeau et al., 2016; Trudeau, Verma, Sampedro, et al., 2017; Trudeau, Verma, Urriola, et al., 2017). Furthermore, the spray drying process also was effective in inactivating infectious PEDV in plasma protein (Gerber et al., 2014). Finally, in regards to pelleting, conditioning and pelleting temperatures above 54.4°C were effective in reducing the quantity and infectivity of PEDV in swine feed (Cochrane et al., 2017). In contrast, viable virus was present following exposure to lower (37.8°C and 46.1°C) conditioning temperatures.

### 2.3 Strategy 3: Chemical mitigation

Extensive studies have been conducted to evaluate the effect of chemical mitigation on PEDV‐contaminated feed (Cochrane et al., 2019; Dee, Neill, Clement, Christopher‐Hennings, & Nelson, 2014; Huss et al., 2017; Trudeau et al., 2016). The initial work revolved around Sal CURB^®^ (Kemin Industries), an FDA‐approved liquid anti‐microbial used to control *Salmonella* contamination in poultry and swine diets. In groups of pigs fed Sal CURB^®^‐treated feed spiked with PEDV versus non‐treated feed, clinical signs of PEDV infection (vomiting and diarrhoea) and viral shedding in faeces were observed 2–3 days post‐consumption of non‐treated feed. In contrast, no evidence of infection was observed in pigs fed Sal CURB^®^‐treated feed (Dee, Neill, et al., 2014). In another study, feed samples were spiked with PEDV and mixed with either organic acid mixtures, sugar or salt and were incubated at room temperature for up to 21 days. All additives tested were effective in reducing the survival of PEDV as compared to non‐treated controls (Trudeau et al., 2016). Recent work by Cochrane and others compared the efficacy of MCFA to other common fat sources to minimize infectivity of feed contaminated with PEDV (Cochrane et al., 2019). Results indicated that feed treated with individual MCFA, 1% MCFA blend or formaldehyde had less detectable viral RNA than other treatments, such as canola oil, coconut oil, palm kernel oil and choice white grease. In addition, PEDV‐contaminated feed treated with formaldehyde, 1% MCFA, 0.66% caproic, 0.66% caprylic and 0.66% capric significantly reduced infectivity, in contrast to feed treated with C12 or longer chain fatty acid sources.

In regards to the elimination of PEDV from a contaminated animal feed manufacturing facility, the combined application of a quaternary ammonium‐glutaraldehyde blend cleaner, followed by a sodium hypochlorite sanitizing solution, along with a facility heat‐up to 60°C for 48 hr was effective at reducing PEDV genomic material, but did not completely eliminate it, demonstrating the residual risk of this virus at the feed mill level following purposeful contamination (Huss et al., 2017).

### 2.4 Strategy 4: Responsible Imports

With the generation of new knowledge on viral half‐life in feed, the application of a ‘Responsible Imports’ approach has been adapted across the US industry (Patterson, Niederwerder, & Dee, 2019). Responsible Imports, a science‐based protocol to safely introduce essential feed ingredients from high‐risk countries using extended periods of storage, is based on the following principles:

**Necessity**: is importation of the ingredient an absolute necessity?
**Alternatives:** can the ingredient be obtained from a country free from foreign animal diseases?
**Virus:** which virus is causing the concern?
**Viral half‐life:** is there published information on the half‐life of the virus in the designated ingredient?
**Transport time:** what is the projected time for delivery of the ingredient from the source to its destination?
**Mitigation:** are there safe products that can be added to the ingredient to reduce viral load during transport?
**Storage period:** is there published information on storage time and temperature that will eliminate residual virus from the ingredient prior to use?

Therefore, as production companies across the United States develop storage facilities for incoming products, a new way of thinking is taking shape, one that is based on ‘feed quarantine’ that brings together information across several disciplines including feed science, microbiology and oceanic transport logistics to understand how to minimize risk. This approach is intriguing as it is non‐regulatory in nature and does not negatively impact trade.

## 3 CONCLUSIONS AND NEXT STEPS

In summary, there is a growing body of scientific evidence suggesting that contaminated feed and feed ingredients purposefully inoculated with viruses may be risk factors for the spread of viral diseases at the domestic and the transboundary levels. This information has stimulated collaborative efforts across North America between livestock and grain commodity groups, governmental agencies, and the veterinary profession in an effort to manage this risk. For example, the Canadian Food Inspection Agency has already implemented a national program using designated secondary control zones to manage the introduction of high‐risk feed ingredients, such as grains, oilseeds and meals from 44 ASFV‐positive countries into Canada. Activity in the United States includes the passage of resolutions from the National Pork Producers Council requesting collaborative efforts across North America to reduce the risk of foreign animal disease entry via the risk of feed ingredients, widespread use of feed additives as chemical mitigants and the writing of policy to guide the implementation of the Responsible Imports approach. A US feed safety task force involving representatives from governmental agencies and multi‐species stakeholder groups has been formed to develop a national plan to manage this risk. Finally, requests to restrict the importation of high‐risk feed ingredients such as soy‐based products from ASFV‐positive countries are being made to key government officials. Collectively, representatives from Mexico, Canada and the United States have begun the discussion of how to collaborate to reduce the risk of the introduction of ASFV and other foreign animal disease pathogens to North America, with focused discussion on all risk factors, including feed.

However, despite all the scientific evidence, there are still differences in opinion as it pertains to the risk of feed. While a recent review concluded that the current body of scientific knowledge lacks conclusive evidence of virus contamination of imported non‐animal origin feed ingredients of commercial swine feed (Gordon et al., 2019), another review concluded that there is a moderate risk for the introduction of ASFV and PEDV to the United States through contaminated feed (Jones, Woodworth, Dritz, & Paulk, 2019). Some reason for this discrepancy is that the latter publication took into account all existing published studies on the transmission of ASFV in feed (Niederwerder et al., 2019), as well as a recent report citing the detection of Seneca Virus A in swine feed and feed ingredients in Brazil (Leme, Miyabe, Dall Agnol, Alfieri, & Alfieri, 2019), while the former paper did not. Clearly, as the body of scientific evidence surrounding the risk of feed continues to grow, the accuracy of risk analyses will improve.

However, despite the progress that has been made, significant research gaps still exist regarding the risk of feed. For example, the vast majority of the published papers are based on experimental inoculation and models. Further efforts to reproduce this work using actual modes of transport, that is actual ocean freighters and commercial transport vehicles trucks, are needed. In addition, it is argued that there is a lack of evidence documenting the presence of viral pathogens in actual feed samples around the world. While the current evidence is indeed limited, viable PEDV has been detected in feed bins feeding index cases of PED on sow farms (Dee, Clement, et al., 2014) and ASFV DNA has been detected in Chinese feed and feed dust from bulk grains stored on the ground post‐harvest, along with samples from feed mills, personnel and delivery vehicles (Proceedings, 1st International Symposium of prevention of ASF, Henan, China, 2019). To compound this problem, a universally validated method to test feed is not available and routine surveillance testing of feed and feed ingredients is not permitted in the United States. Furthermore, there are no feed additives that are currently approved by the US Food and Drug Administration to mitigate the risk of viral‐contaminated feed. Fortunately, research is ongoing to identify additional mitigant candidates and conversations are underway between feed companies and government officials regarding the approval process.

In closing, in a few short years, global agriculture has come a long way in recognizing and accepting the risk of feed and feed ingredients as vehicles for the domestic and transboundary spread of diseases, based on the research efforts cited in this writing. It is hoped that these efforts will continue to stimulate communication and collaboration between the feed and livestock industries, resulting in further research into the emerging concept of ‘global feed biosecurity’. Ideally, current and future information regarding the risk of pathogen spread in feed will enhance the accuracy of risk assessments, drive the continual development of efficacious feed‐based mitigation strategies and ultimately change the philosophy regarding the global trade of feed ingredients from one that based on price to one where the biosecurity of the feed supply chain is prioritized.

## CONFLICT OF INTEREST

The authors declare no conflict of interest.

## ETHICAL APPROVAL

No ethical approval was required as this is a review article with no original research data.

## Data Availability

All references used to write this review were disclosed.
